# An Image Analysis Solution For Quantification and Determination of Immunohistochemistry Staining Reproducibility

**DOI:** 10.1097/PAI.0000000000000776

**Published:** 2019-05-06

**Authors:** Elizabeth A. Chlipala, Christine M. Bendzinski, Charlie Dorner, Raili Sartan, Karen Copeland, Roger Pearce, Faye Doherty, Brad Bolon

**Affiliations:** * Premier Laboratory LLC; § GEMpath Inc., Longmont; ‡ Boulder Statistics LLC, Steamboat Springs, CO; † Dako (an Agilent Technologies Company), Carpinteria, CA

**Keywords:** immunohistochemistry, reproducibility, image analysis, accordance, accuracy, precision, assay validation

## Abstract

With immunohistochemical (IHC) staining increasingly being used to guide clinical decisions, variability in staining quality and reproducibility are becoming essential factors in generating diagnoses using IHC tissue preparations. The current study tested a method to track and quantify the interrun, intrarun, and intersite variability of IHC staining intensity. Our hypothesis was that staining precision between laboratory sites, staining runs, and individual slides may be verified quantitatively, efficiently and effectively utilizing algorithm-based, automated image analysis. To investigate this premise, we tested the consistency of IHC staining in 40 routinely processed (formalin-fixed, paraffin-embedded) human tissues using 10 common antibiomarker antibodies on 2 Dako Omnis instruments at 2 locations (Carpinteria, CA: 30 m above sea level and Longmont, CO: 1500 m above sea level) programmed with identical, default settings and sample pretreatments. Digital images of IHC-labeled sections produced by a whole slide scanner were analyzed by a simple commercially available algorithm and compared with a board-certified veterinary pathologist’s semiquantitative scoring of staining intensity. The image analysis output correlated well with pathology scores but had increased sensitivity for discriminating subtle variations and providing reproducible digital quantification across sites as well as within and among staining runs at the same site. Taken together, our data indicate that digital image analysis offers an objective and quantifiable means of verifying IHC staining parameters as a part of laboratory quality assurance systems.

## Abstract 2

Supplemental Digital Content is available in the text.

Immunohistochemistry (IHC) has become a common tool in clinical and research laboratories as a more specific molecular pathology alternative to routine histochemical staining. In research, immunodetection of specific biomarkers between treatment groups can be correlated to other types of expression data. In the clinical realm, IHC is a facile way to quickly visualize the presence of biomarkers in the context of tissue morphology and has gained traction as a routine diagnostic aid for many diseases, notably breast cancer.^1^–^9^ The clinical use of IHC is considered by the US Food and Drug Administration as a laboratory-developed, “cell-based in situ immunoassay” rather than a histochemical stain. Thus, effective quality assurance (QA) steps are needed to validate such tests for clinical use.^10^–^12^

Recent efforts to standardize IHC for clinical use have emphasized proper protocol validation due to the historically poor repeatability of IHC assays in nonclinical studies.^13^ Part of the IHC standardization process in the clinical setting is the evaluation of test performance characteristics (TPCs) for a particular IHC protocol. The TPCs discussed in this paper are related to the staining process itself (ie, analytic) rather than preparation of the samples to be stained (preanalytic).^14^,^15^ The reason for this focus is that relatively more is known about the impact of preanalytic parameters on IHC labeling quality, whereas comparatively little has been published with respect to the effect of analytic factors on the reproducibility of immunoassays.

Three TPCs that this study addresses are sensitivity, reproducibility, and precision.^11^,^12^,^16^ Sensitivity describes the lowest level of detection achieved by an IHC protocol. Sensitivity is determined by staining a tissue that is expected to have low expression of the target and evaluating the resultant signal. Reproducibility describes the consistency of results among tests (eg, the likelihood of all positive tests correctly detecting a positive result, or vice versa), and precision describes the degree to which multiple repetitions of an assay yield the same result. Two aspects need to be addressed when evaluating the reproducibility of an assay: agreement between laboratories, and agreement within a laboratory. As gauges of reproducibility and precision, accordance is defined as the percentage agreement between laboratories for results obtained from identical specimens, whereas concordance (which is analogous to precision) is defined as the percentage agreement of results for repeated analysis of samples within a given facility.^17^ Some researchers argue that precision cannot be applied to IHC data because such assays are qualitative by nature.^12^ This study was designed to investigate the proposition that IHC data acquired from tissue sections may be quantified with sufficient rigor across staining runs and laboratories to permit both accordance and concordance to be measured in a way that falls under the realm of true “precision.”

The goal of the present study was to assess the sensitivity, reproducibility, and precision of various IHC assays in a quantitative manner using automated image analysis. Multiple human tissue types with a range of antigen expression levels were used for each antibody, effectively allowing for quantification of staining sensitivity, whereas interrun and intrarun repetitions tested the reproducibility and precision of staining. The authors hypothesized that both the precision and reproducibility of IHC assays are amenable to quantifiable measurement by automated image analysis. To the authors’ knowledge, this study is the first that has assessed the utility of automated image analysis to investigate TPCs using a large range of targets for runs on equivalent instruments located at separate laboratories in distinctly different planetological zones. With the rapidly growing significance of IHC-based diagnostic assays in the clinical setting, it is imperative that laboratories devise ways to effectively monitor staining quality of their own IHC output. Our current data show that the use of automated image analysis presents an efficient, effective, low-cost method for tracking the variability of IHC staining at any given instant and over time. Our data also suggest that implementing an algorithm-based automated image analysis system may be able to reduce the delays and expense associated with current requirements that an experienced pathologist be involved routinely in the quality control and day-to-day analysis of IHC-stained sections.

## MATERIALS AND METHODS

### Laboratories

Two distant facilities participated in this study. The Dako site was located near sea level (elevation<30 m) in Carpinteria, CA, whereas the Premier Laboratory LLC site was located at high altitude (elevation of 1500 m) in Longmont, CO. The choice of 2 different altitudes is of great relevance to this project, as it has been shown that the heat-induced epitope retrieval procedure inherent to the staining procedure is affected by high altitude for some IHC markers.^18^

### Reagents

Ten well-characterized antibodies [AE1/AE3, BCL2, CD20, CD3, CD68, cytokeratin 7, E-cadherin, Ki-67, MSH2, and synaptophysin (Dako, Carpinteria, CA)] were selected to represent a range of nuclear, cytoplasmic, and membrane staining patterns in a variety of tissues (Table 1). All antibodies were in the ready-to-use formulation, and all other Dako reagents [heat-induced epitope retrieval solutions, buffers, labeled polymer, 3,3′-diaminobenzidine (DAB) and hematoxylin counterstain] were designed for use with the Dako Omnis Autostainer. The same antibody solutions were used across all 12 staining runs at 2 locations, and all staining runs were performed within 21 days. All reagents for all staining runs were from single lots.

**TABLE 1:**
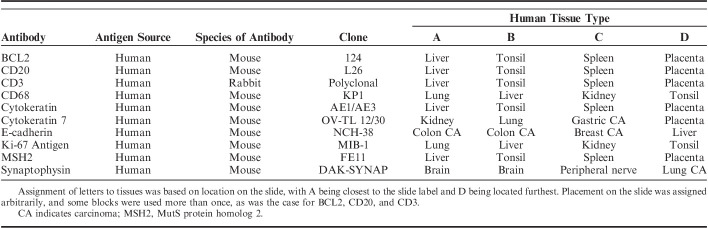
Antibodies and Tissues Used

### Tissues and Slides

Neutral buffered 10% formalin-fixed, routinely processed, paraffin-embedded human tissues (4/antibody) were selected to represent a range of anticipated levels of antigen expression, and therefore, staining intensities.^19^–^21^ Tissues were either purchased specifically for this study or sampled from the control tissue inventory at Premier Laboratory LLC; the length of time in fixative was unknown. The 4 tissues for each antibody were embedded in a multitissue block in an arbitrary order (Fig. S1, Supplemental Digital Content 1, http://links.lww.com/AIMM/A236), and 40 serial sections per block were cut by Premier Laboratory LLC at 4 μm and each placed in the center of FLEX coated slides (Dako) to promote section adhesion. Adjacent sections were used for each comparison (odd-numbered sections for runs at the sea level laboratory and even-numbered ones for those in the high-altitude facility). For each antibody, 6 runs with 3 slides each were performed at each location (36 slides total). Slides were air-dried and baked at 60°C for 20 to 60 minutes before IHC staining.

### Immunohistochemical Staining

Two equivalent Dako Omnis Autostainers (Dako) designed for combined IHC and in situ hybridization procedures were used. All staining steps for all 10 antibody assays were automated, including hematoxylin counterstaining. Default IHC protocols from the Omnis instrument software were used according to the manufacturer’s instructions for all staining runs at both laboratories.

### Quantitative Automated Image Analysis

Stained slides were scanned at 20X magnification using the Aperio ScanScope XT imaging system (Aperio, Vista, CA) and analyzed using the accompanying ImageScope software. Each tissue section (4/slide) was annotated using an 8000×4000-pixel area bounding box (Fig. S1, Supplemental Digital Content 1, http://links.lww.com/AIMM/A236). Efforts were made to select the same or similar tissue areas across all 36 slides per antibody. The annotated areas were analyzed using a modified DAB-specific positive pixel count algorithm, differentiating each pixel into negative (n), weak positive (wp), positive (p), or strong positive (sp) bins (Fig. S2, Supplemental Digital Content 1, http://links.lww.com/AIMM/A236). Each group was defined by a threshold of intensity values [*I*_n_=(220,255); *I*_wp_=(175,220); *I*_p_=(100,175); *I*_sp_=(0,100) for n, wp, p, and sp bins, respectively]. The magnitudes of threshold values indicate an inverse relationship between stain darkness and pixel intensity, where negative (unstained) pixels are of high intensity while strong positive pixels are of low intensity.

Quantitative image analysis was performed using the average intensity of positive pixels (*I*_avg_), the relative number of strong positive pixels (*N*_sr_), and the percent positive pixel count. *I*_avg_ was calculated by adding the intensities of every positive pixel and dividing by the total number of positive pixels [*I*_avg_=(*I*_wp_+*I*_p_+*I*_sp_)/(*N*_wp_+*N*_p_+*N*_sp_)]. *N*_sr_ was calculated by dividing the number of strong positive pixels by the total number of positive pixels [*N*_sr_=*N*_sp_/(*N*_wp_+*N*_p_+*N*_sp_)].

### Semiquantitative Histopathology Evaluation

All 36 stained slides for 3 antibodies from each of 6 tissues were reviewed by a board-certified veterinary anatomic pathologist. The 3 antibodies and 6 tissues were selected by choosing those with the most variable staining intensity as determined by prior automated image analysis. The combinations of antibodies and tissues were: AE1/AE3 on liver, tonsil, and placenta; CD20/L6 on tonsil and spleen; and Ki-67 on tonsil. Each section was assigned a score from 0 to 5 based on DAB labeling intensity (Table 2) using a coded (blind) histopathologic evaluation strategy.

**TABLE 2:**
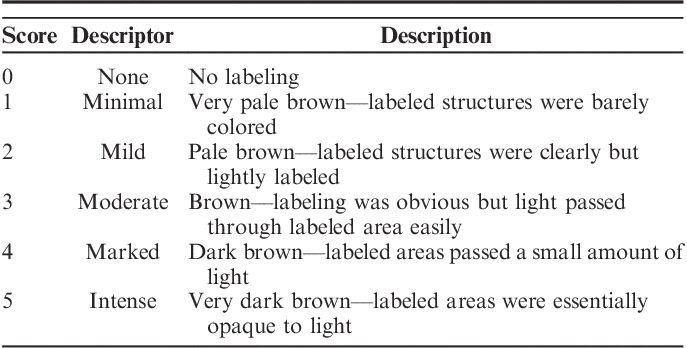
Pathologist Scoring Criteria

### Statistical Analysis

The measurement system was evaluated using nested variance component models in commercially available statistical software (JMP Pro 14.2; SAS Institute, Cary, NC) according to the manufacturer’s instruction. These models were used to quantify the intrarun variability, interrun variability, and intersite variability for the relative number of strong positive pixels (*N*_sr_) and average intensity of positive pixels (*I*_avg_). The variance components are expressed as a percentage of the coefficient of variation (% CV, calculated as the SD/mean) and summarized across the 40 tissues (4 tissues×10 antibodies; n=36 for each sample). A bivariate analysis was used to evaluate the correlation between *I*_avg_ and *N*_sr_ for all tissues and antibodies and to assess the degree of correlation between data derived from the automated image analysis and histopathology evaluation. To eliminate noise in the latter analysis, 19 CD20-stained samples were excluded (N=197; Fig. S3, Supplemental Digital Content 1, http://links.lww.com/AIMM/A236). An *F* test for unequal variance was used to compare variances between sites for each antibody and tissue combination. To account for running 40 tests of significance, the threshold for significance was set to *P*-value <0.001.

## RESULTS

**FIGURE 1:**
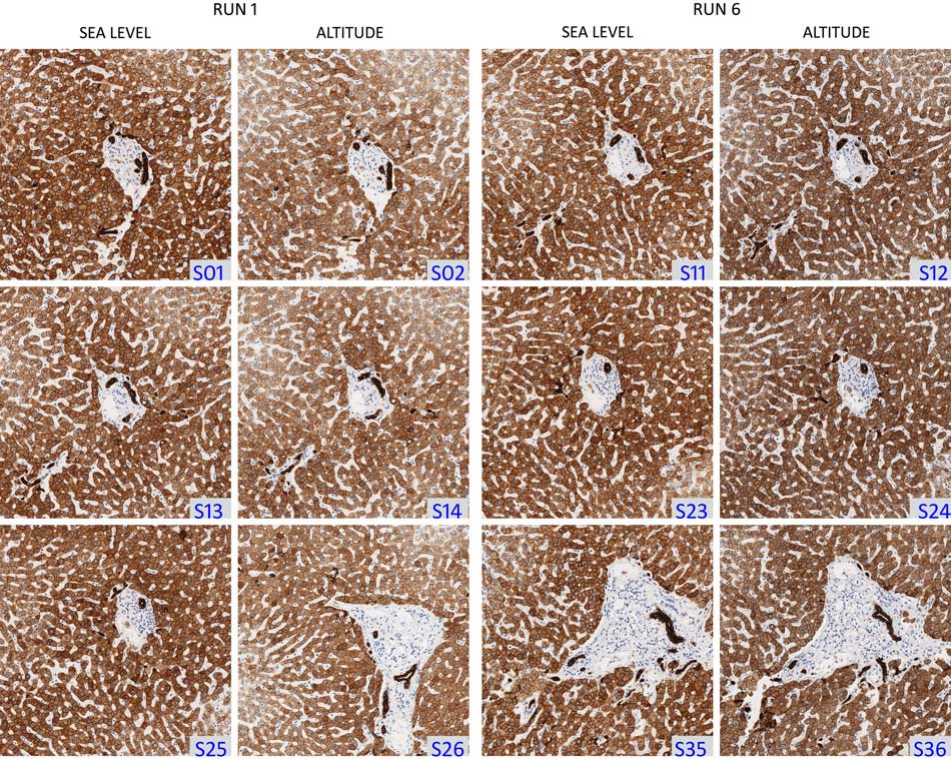
Representative photomicrographs of cytokeratin (AE1/AE3) staining in human liver. Triplicate slides were stained with anti-AE1/AE3 cytokeratin antibody in 12 separate staining runs across 2 distant locations (“sea level”: Carpinteria, CA; “altitude”: Longmont, CO). Only the first and last runs at each location are pictured, showing triplicates from 4 separate representative runs. Cytokeratin was visualized using 3,3′-diaminobenzidine (brown) with hematoxylin counterstain (blue). S## indicates sequentially-numbered serial sections.

No systematic differences were observed in staining intensity between the 2 Dako Omnis instruments at sea level and altitude, based upon positive pixel count image analysis. Tissue A (liver) stained with anti-AE1/AE3 showed more variability in *I*_avg_ (7.7% CV overall and 4.5% CV between locations) than 75% of the 40 tissues tested, across all antibodies. However, these differences were subtle, and appeared minor to the naked eye (Fig. 1). Histopathology assessment of AE1/AE3-stained liver tissue showed that slides stained at sea level were more intensely stained on average (mean score, 3.1) than their counterparts at altitude (mean score, 2.5).

**FIGURE 2:**
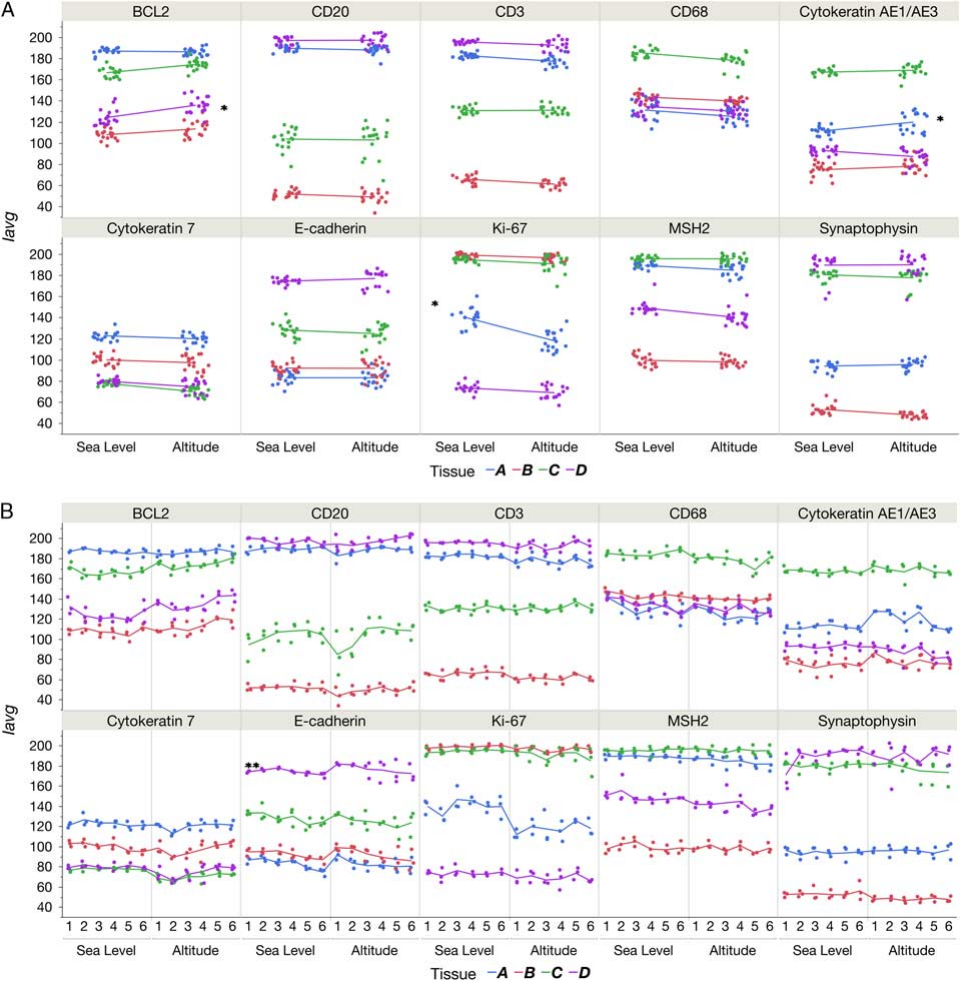
Staining reproducibility. A, Comparison of staining intensities between 2 equivalent instruments in distant locations. Average positive pixel intensity (*I*_avg_) was calculated for each image from the data output from the modified positive pixel count algorithm as predefined in the automated analysis software. Tissue type for each antibody is differentiated by color (where tissue A=blue, tissue B=red, tissue C=green, and tissue D=violet); for exact tissue identification organized by antibody used, see Table 1. For each antibody, 18 slides per tissue were stained at each location (“sea level”: Carpinteria, CA; “altitude”: Longmont, CO), which generated clusters of 18 data points each. The mean for each data cluster between the 2 sites was connected by a trendline. A flat line shows high intersite reproducibility, whereas a heavy slant indicates lower reproducibility between sites. B, Comparison of average positive pixel intensities (*I*_avg_) of replicates across all runs and locations. Tissue type is differentiated by color (where tissue A=blue, B=red, C=green, D=violet); for exact tissue identification organized by antibody, see Table 1. Each run is plotted as a separate vertically stacked group of data points, showing intrarun variability. The vertical gray lines separate locations (“sea level”: Carpinteria, CA; “altitude”: Longmont, CO). The mean *I*_avg_ for each run is connected by lines; level lines indicate interrun consistency, and vertical space between data points in each column indicate intrarun consistency.

Intersite variability was compared graphically by connecting the mean *I*_avg_ between sites for each tissue across all antibodies. For a minority of tissues, the mean *I*_avg_ changed between staining at sea level and altitude (indicated by sloped lines in Fig. 2A). For example, the steep slopes for the different locations in the cases of Ki-67 tissue A (lung, blue line), BCL2 tissue D (placenta, violet line), and AE1/AE3 tissue A (liver, blue line) were all indicative of high intersite variability (12.1%, 6.1%, and 4.5% CV, respectively, indicated with single asterisks in Fig. 2A). For a given antibody, staining consistency within and among runs was fairly consistent for a given location and across the 2 locations (Fig. 2B). However, in certain instances the intrarun variability was higher for some antibodies, especially when stained at altitude. For example, E-cadherin staining of Tissue D showed a high intrarun variability (CV of 5.4%, 2 asterisks in Fig. 2B) for a number of runs at altitude, which was undiscernible using the clustered graphical representation in Figure 2A. In addition, CD20 staining in tissue C was highly variable both between and within runs but showed similar results at each location (Fig. 2B).

**FIGURE 3:**
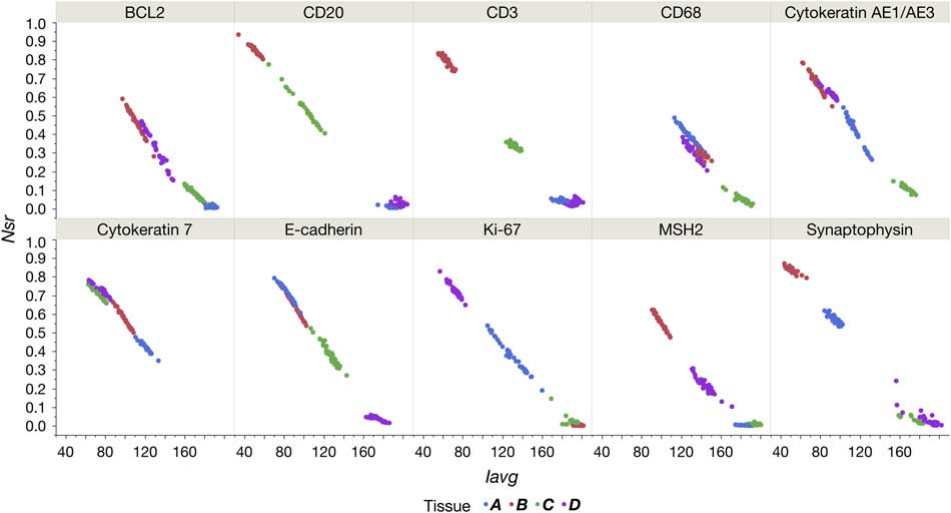
Correlation of relative number of strong positive pixels (*N*_sr_) and average staining intensity of positive pixels (*I*_avg_). A bivariate analysis compared *N*_sr_ and *I*_avg_ for both locations and all tissues for each antibody. Each panel plots the (*I*_avg_, *N*_sr_) points for each of tissues A–D (by color), for both locations (“sea level”: Carpinteria, CA; “altitude”: Longmont, CO) combined, for that specific antibody. Tissue type is differentiated by color (tissue A=blue, B=red, C=green, D=violet); for exact tissue identification organized by antibody used, see Table 1.

To maintain consistency between quantitative (automated image analysis) and semiquantitative (histopathology) scoring standards, the correlation between the relative number of strong positive pixels (*N*_sr_) and average intensity of positive pixels (*I*_avg_) was examined (Fig. 3). The 2 metrics were strongly negatively correlated, as a visually dark stain had a low intensity value but typically a high percentage of positive pixels.

**FIGURE 4:**
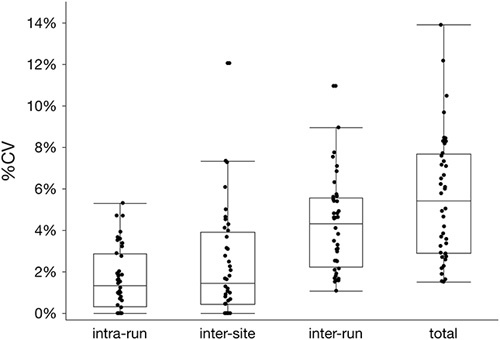
Variance components for all tissues, antibodies, and sites. Nested variance component models were used to quantify the interrun, intrarun, and intersite variability as well as the total variability across all samples. The components were separated into categories, from left to right: “intrarun,” variability among the 3 slides in each run; “intersite,” between locations; “interrun,” among runs regardless of location; and total variability overall. Each data point represents the % CV for that component for each tissue (reflecting 4 tissues per antibody for 10 antibodies, or N=40 for each component/point). The box plots outline the range from the 25% quantile to 75% quantile (interquartile range), the horizontal line within each box represents the median, and error bars denote the 90% confidence interval.

To quantify the intrarun, intersite, interrun, and total variability, nested component variance models were constructed for each antibody, and then compared across the 10 antibodies (1440 individual samples; Fig. 4, Table S1, Supplemental Digital Content 2, http://links.lww.com/AIMM/A237). Splitting the data into components allowed for each variable (intersite, intrarun, or interrun) to be quantified with respect to how much its variability contributed to the total variability. The component with the largest interquartile range (distance between the 25% and 75% quantiles) was location (ie, intersite), with a spread of 3.46% CV. The intrarun component had the smallest variability (2.55% CV interquartile range). In other words, the site-to-site differences contributed the most to the total variability in the results, whereas slide-to-slide differences within a single run contributed the least. This intersite variability was examined by an *F* test for unequal variances for all 40 tissue and antibody combinations (Table 3). Although 12 of these tests showed unequal variance between sites, no tissue showed consistent difference in variance across all stains. Similarly, no stain showed consistent differences across all 4 tissues. For instances in which a difference in variance was observed, samples stained at altitude showed a higher variance than those at sea level, with 1 exception (tissue B, synaptophysin). Overall, this study does not support a systematic difference in *I*_avg_ variability due to the site location.

**TABLE 3:**
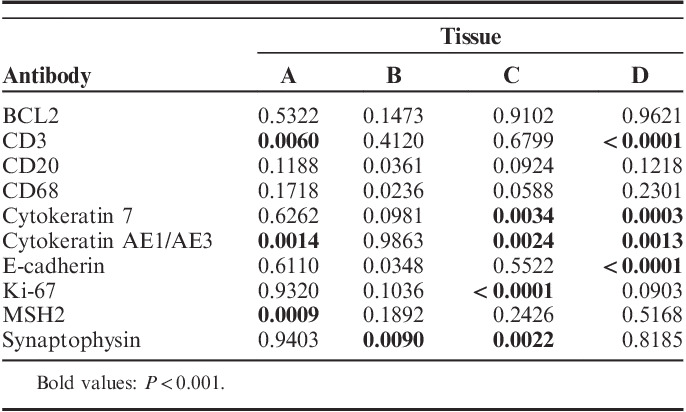
*P*-Values for *F* Tests For Unequal Variance of *I*_avg_ For 40 Tissue/Antibody Combinations

**FIGURE 5:**
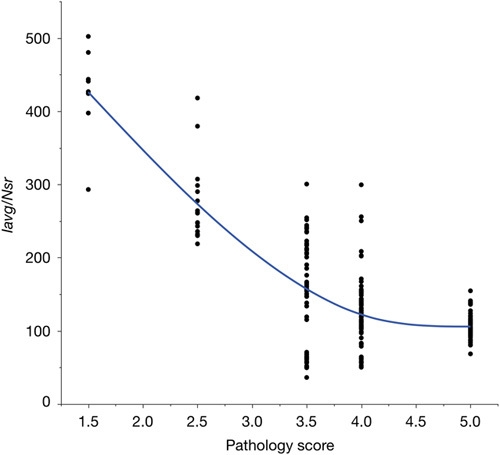
Comparison of quantitative automated image analysis (ratio of *I*_avg_ to *N*_sr_) and semiquantitative histopathology scoring methods. A nonlinear, negative relationship between these variables is observed, demonstrating a good degree of agreement between them. A total of 216 data points was gathered, but the graph only shows N=197 as 19 data points (8.8%, all from CD20-stained tissues) did not follow the typical correlation between *I*_avg_ and *N*_sr_ (as is illustrated in Fig. S3, Supplemental Digital Content 1, http://links.lww.com/AIMM/A236).

Histopathology scores obtained by coded (blind) evaluation were compared with the automated image analysis output for both the average intensity of positive pixels (*I*_avg_) and the number of strong positive pixels (*N*_sr_). Each histopathology score spanned a large range of *I*_avg_ and *N*_sr_ values. To take both image analysis metrics into account, the ratio of *I*_avg_ to *N*_sr_ was compared with histopathology scores in a bivariate analysis (Fig. 5). These 2 variables were related by a nonlinear, negative relationship, demonstrating an overall agreement between the 2 methods.

## DISCUSSION

The growth of IHC methods as research tools and clinical diagnostic assays has led to an urgent need to devise new means for unbiased evaluation of IHC staining. Traditionally, such interpretations have been performed by pathologists using semiquantitative scoring schemes that evaluate staining intensity (light and dark) and/or the extent of staining (area covered).^18^ With the advent of computer-based platforms, fully quantitative automated image analysis software with predefined “turn-key algorithms” allows simultaneous validation of staining area and staining intensity by researchers equipped with commercially available instruments.^14^,^15^ The expanding use of automated image analysis systems to acquire IHC data requires that practitioners be prepared to appropriately perform QA monitoring for their IHC assays.

The present study investigated the intersite concordance between identical automated immunostainers housed at 2 analytical locations in different geographic settings (high altitude and sea level) as well as the interrun and intrarun accordance to detect the precision and reproducibility of IHC staining. Our hypothesis was that staining precision associated with these variables may be verified efficiently and effectively utilizing quantitative predefined algorithms in commercial image analysis software. Our data showed that automated digital image analysis facilitated performance monitoring for IHC assays established for multiple antibodies, providing reliable and reproducible quantitative assessments of precision and sensitivity. In addition, automated analysis diminished (though did not fully replace) the need for skilled histopathology support to provide QA for IHC staining runs.^22^,^23^ Finally, automated image analysis afforded a more sensitive means for detecting nuanced changes in IHC staining, especially at low staining levels at which the human eye tends to have difficulty in discriminating subtle variations in color intensity.^24^,^25^

Overall, the observed IHC staining intensity on equivalent instruments at sea level (<30 m) and high altitude (1500 m) showed a similar degree of variation to that seen among runs on the same instrument. The total variability was largely affected by differences in tissue morphology resulting from the distance between sections taken from the same block, rather than from variations in staining quality inherent to the IHC method. The intrarun variability, and thus total variability, could be reduced by a change in the study design that uses serial sections for all variables. In this case, each variable would be studied separately off the same block, and morphologic differences among sections would likely contribute much less to the total variability.

Scores given by the pathologist (with 30 y of experience in evaluating IHC staining) generally agreed with the automated image analysis output for average intensity of positive pixels (*I*_avg_) values. That said, the automated system was better able to sensitively discriminate subtle nuances in faintly-stained sections. This observation suggests that detection of long-term, subtle trends affecting IHC staining quality associated with a commonly performed IHC assay might easily be accomplished with automated image analysis, which would prove useful to research and clinical laboratories alike.

The determination of intersite reproducibility is critical for the standardization of IHC protocols as performed in highly regulated clinical settings. Although the current study was done with a closed design (reagents and instrument settings kept consistent across assays at 2 separate sites using identical stainers), the reality is that many laboratories use open systems (nonstandard reagents and dissimilar instruments) that automatically will introduce more variability. Nonetheless, our current data provide guidance regarding the sorts of QA analysis that might provide support for the precision and reproducibility of IHC data. Therefore, any laboratory, clinical or research, should implement measures to monitor IHC staining quality, especially when the slides are to be used in prognostic and therapeutic decision-making. An obvious corollary concept is that utilization of a subjective “pass/fail” method in evaluating the validity of staining runs should be abjured in favor of the more quantitative and reproducible results that can be obtained by automated analytical protocols.^14^,^26^ An important consideration in migrating to automated analysis of IHC data is the ease with which such measurements may be undertaken. Our current automated analysis showed that the nested variance component models present as predefined options in the software package are quite helpful to verify staining consistency and reproducibility across many different variables while requiring little computer sophistication in terms of software coding.

Looking forward, other options that were not explored here could be investigated as a means of improving IHC reproducibility. For instance, the bounding box used in the automated image analysis could be eliminated as the recent improvement of algorithms and the use of tissue optical densities has allowed for increasingly effective automatic selection of individual tissue types and cell populations. Similarly, the negative relationship between intensity and perceived darkness of staining (ie, dark staining corresponds to a low intensity value) is confusing and suggests that the use of optical density^21^,^27^ (absorbance divided by intensity) may be a more intuitive way to describe what the observer sees. Although number of strong positive pixels (*N*_sr_) had a positive correlation with histopathology scores, *N*_sr_ is merely a pixel count within a threshold range rather than a quantification of the intensity of all pixels, which makes the true relationship between histopathology scores and *N*_sr_ equivalent to comparing apples to oranges.

Although the goal of this study was largely to present a sensitive and unbiased method for IHC quality control, many applications of IHC suggest and reinforce the need for regular input by an experienced histopathologist. A poorly calibrated algorithm that is not tuned to clinically relevant structures, for example, will limit the usefulness of the automated QA procedures we describe here. The complexity of the human brain and its ability to pull from a library of past experiences remains invaluable to the clinical decision-making process.^28^ However, the cost and time-effectiveness of automated image analysis to achieve a high-quality outcome relative to these same parameters when the work is done by a less skilled or experienced technician shows promise for the widespread uptake of this method that will drive a greater awareness of IHC reproducibility across laboratories everywhere.

## Supplementary Material

SUPPLEMENTARY MATERIAL

Supplemental Digital Content is available for this article. Direct URL citations appear in the printed text and are provided in the HTML and PDF versions of this article on the journal's website, www.appliedimmunohist.com.
