# Supplementary figures and images for: An Image Analysis Solution For Quantification and Determination of Immunohistochemistry Staining Reproducibility
Source: Appl Immunohistochem Mol Morphol. 2019 May 6;28(6):428–36. doi: 10.1097/PAI.0000000000000776 (PMC7368846; doi:10.1097/PAI.0000000000000776)

Figure 1


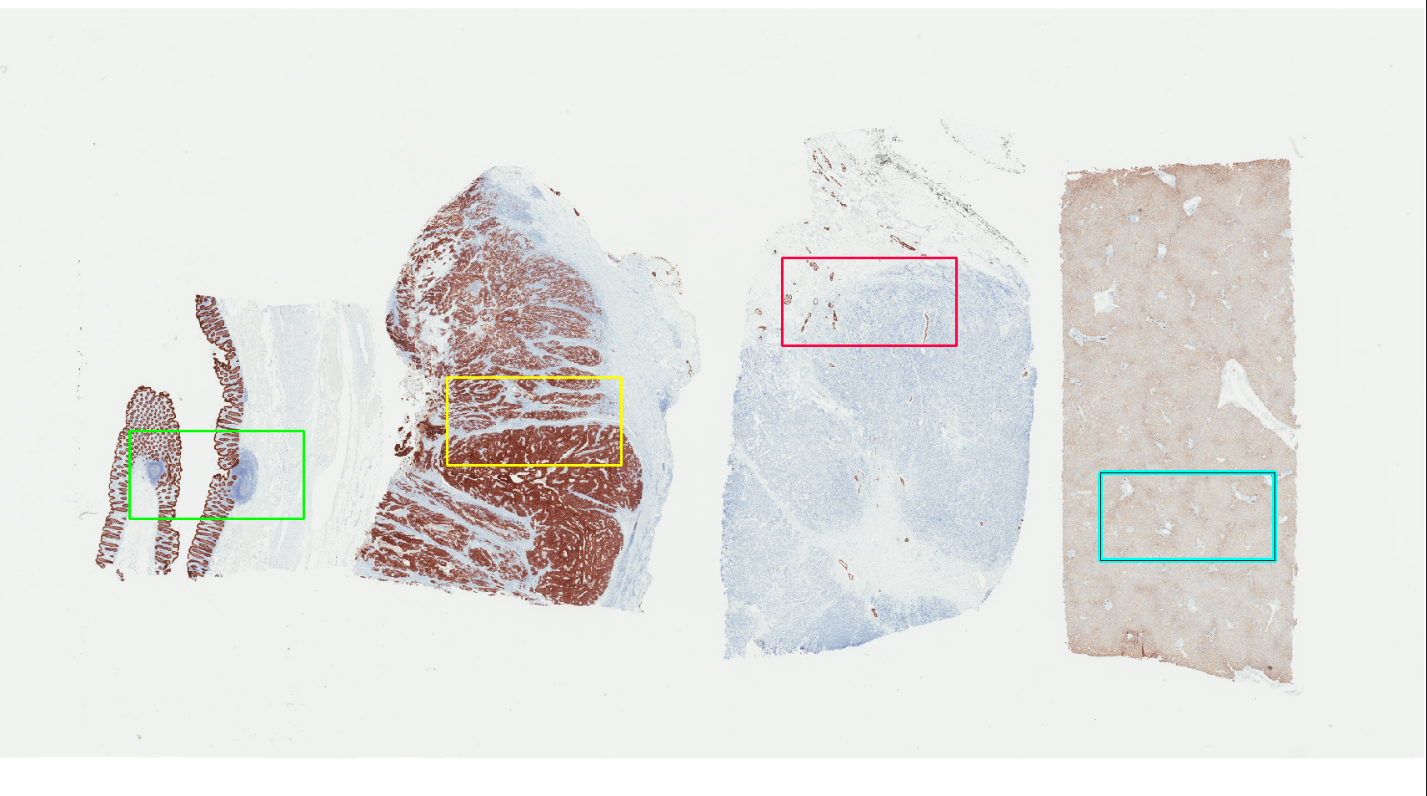


Figure 2a


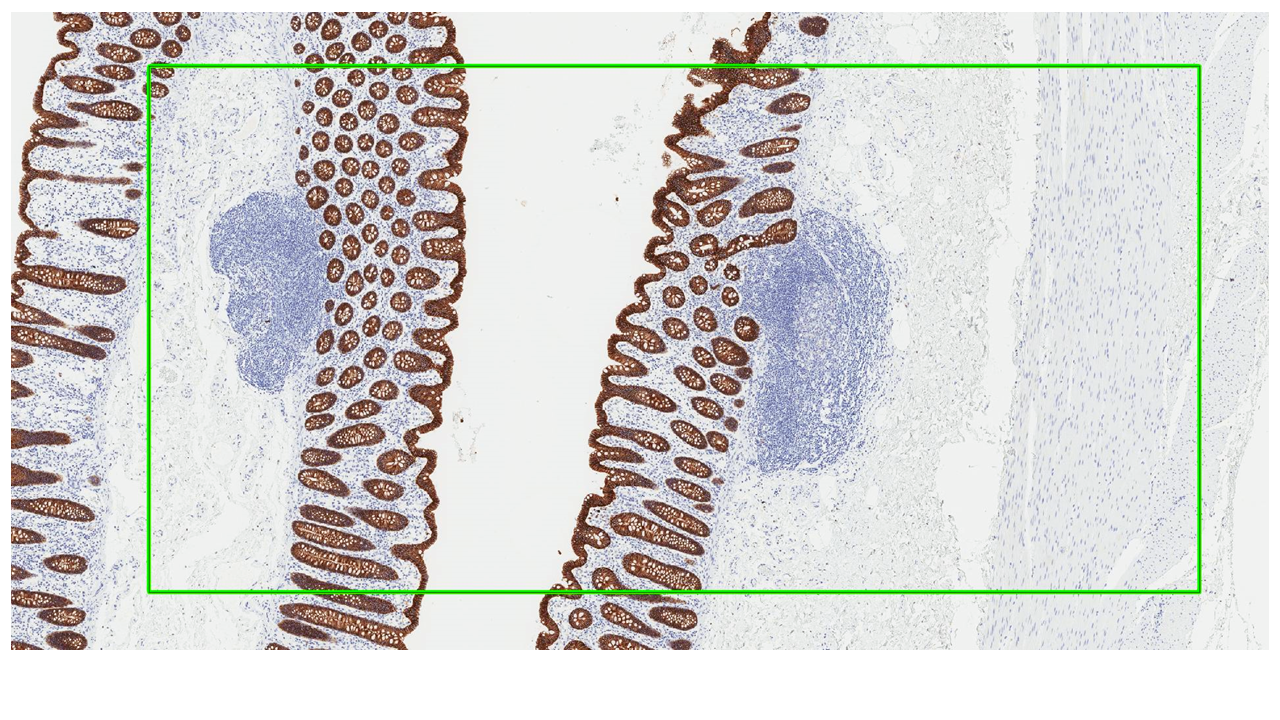


Figure 2b


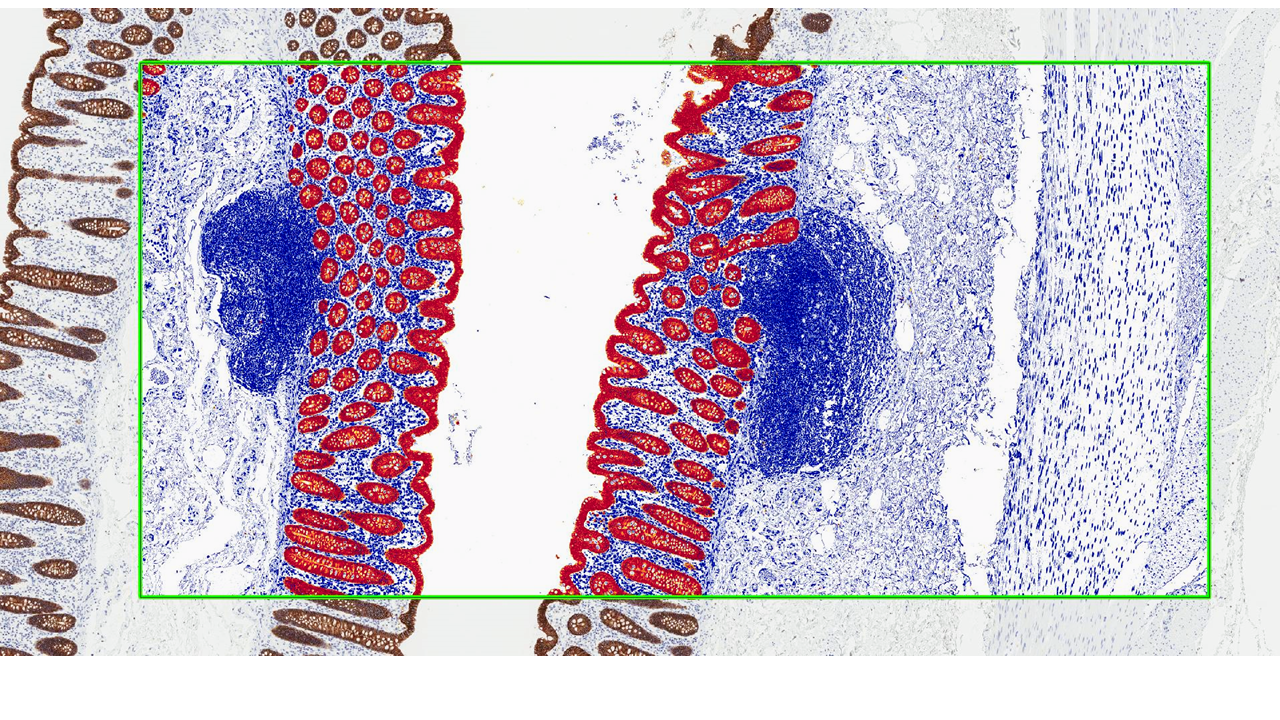


Figure 3


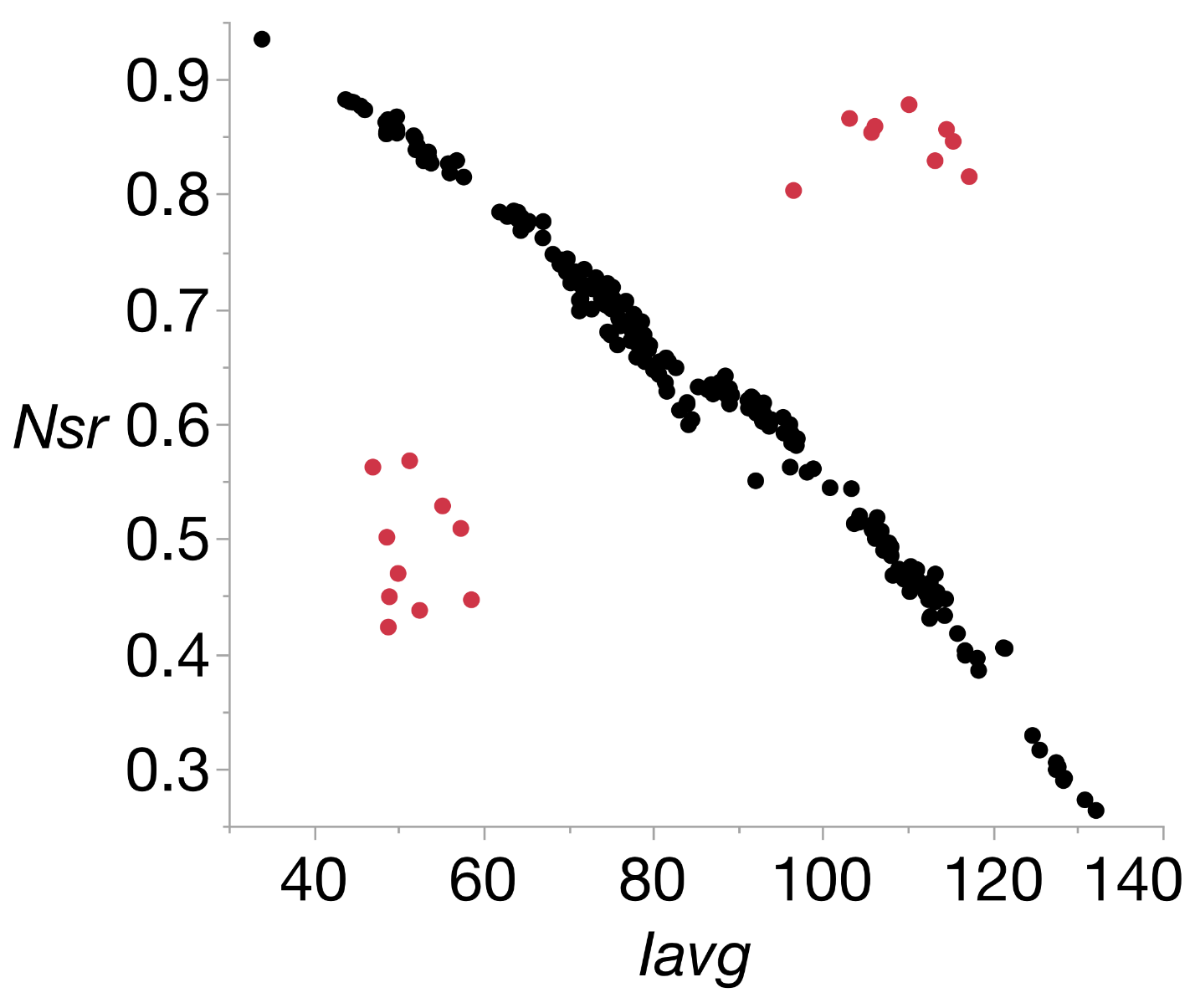

Supplement: SUPPLEMENTARY MATERIAL [file pai-28-428-s001.docx]
