# Supplementary material for: An Image Analysis Solution For Quantification and Determination of Immunohistochemistry Staining Reproducibility
Source: Appl Immunohistochem Mol Morphol. 2019 May 6;28(6):428–36. doi: 10.1097/PAI.0000000000000776 (PMC7368846; doi:10.1097/PAI.0000000000000776)
Supplement: SUPPLEMENTARY MATERIAL [file pai-28-428-s002.docx]

Supplemental Table 1. Nested variance components (% CV) across all 40 tissues for all 10 antibodies.

| **Level** | **Minimum** | **10%** | **25%** | **Median** | **75%** | **90%** | **Maximum** |
| --- | --- | --- | --- | --- | --- | --- | --- |
| **Inter-run** | 1.08% | 1.59% | 2.24% | 4.34% | 5.58% | 7.50% | 10.95% |
| **Intra-run** | 0.00% | 0.00% | 0.31% | 1.34% | 2.86% | 3.90% | 5.31% |
| **Inter-site** | 0.00% | 0.00% | 0.45% | 1.46% | 3.91% | 5.97% | 12.05% |
| **Total** | 1.52% | 1.92% | 2.89% | 5.41% | 7.68% | 9.56% | 13.90% |

% CV = percent coefficient of variance.
